# Prevalence and antibiotic susceptibility patterns of bacteria causing surgical site infections in a tertiary care centers in Lebanon

**DOI:** 10.1371/journal.pgph.0005783

**Published:** 2026-01-07

**Authors:** Inass Kawtharani, Ghassan Ghssein, Hussein Mcheimeche, Mariam Hassan, Pascale Salameh, Najwane Sadier

**Affiliations:** 1 Lebanese University, Ecole Doctorale des Sciences et Technologies, Hadath, Lebanon; 2 Laboratory Sciences Department, Faculty of Public Health, Islamic University of Lebanon, Khalde, Lebanon; 3 Faculty of Sciences, Lebanese University, Beirut, Lebanon; 4 High Council for Scientific Research & Publication (HCSRP), Islamic University of Lebanon, Khalde, Lebanon; 5 Department of Surgery, Al Zahraa Hospital University Medical Center, Beirut, Lebanon; 6 Sahel general Hospital, Beirut, Lebanon; 7 Faculty of Pharmacy, Lebanese University, Beirut, Lebanon; 8 School of Medicine, Lebanese American University, Byblos, Lebanon; Institut National de Santé Publique d’Épidémiologie Clinique et de Toxicologie-Liban, Beirut, Lebanon; 9 Department of Primary Care and Population Health, University of Nicosia Medical School, Nicosia, Cyprus; 10 Public Health and Protection Department, Dubai Health Authority, Abu Dhabi University, United Arab Emirates; 11 Neuroscience research center, Faculty of Medical Sciences, Lebanese University, Hadath, Lebanon; University of Oslo Faculty of Medicine: Universitetet i Oslo Det medisinske fakultet, NORWAY

## Abstract

Surgical Site Infections (SSIs) are a significant global health concern, especially after noticing the development of bacteria that are also resistant to several antibiotics. In Lebanon, data on SSIs and their associated antimicrobial resistance patterns are scarce, highlighting a critical gap in local epidemiological knowledge. This study aimed to determine the distribution of bacterial pathogens and evaluate their antibiotic susceptibility among patients with SSIs in various Lebanese hospitals. The research is a multi-center and prospective cross-sectional study in which data was obtained from patients who developed SSIs after surgical procedures within the period of January – September 2024. Swabs from wounds or tissue samples were taken from the patients, while the isolation and identification of bacteria was performed using standard microbiological techniques through culture on media and biochemical identification tests. The antimicrobial resistance profiles were performed using disk diffusion method. Sociodemographic and medical data was collected in the patients’ records. The Data was analyzed using SPSS. In total, 6933 patients were admitted in the surgical departments of different hospitals. SSIs occurred in 63 patients, with a rate of 0.91%, 95% CI [0.70%, 1.15%]. Gram-negative bacteria predominated (46 (73%)) including *E. coli* (13(20.6%)) and *Pseudomonas aeruginosa* (12 (19%)), compared to Gram-positive bacteria (17 (27%)) such as *Staphylococcus aureus* (8(12.7%)). High levels of antibiotic resistance were found in Gram-positive isolates (71%) and Gram-negative isolates (61%), indicating a significant presence of multidrug-resistant organisms which is a serious threat to public health. This study highlights the high prevalence of antibiotic-resistance in bacteria causing SSIs in Lebanese hospitals and underscores the urgent need for stringent infection control and improved antibiotic management.

## Introduction

Globally, healthcare providers face significant challenges in managing surgical site infections (SSIs), which can result in life-threatening complications, particularly after surgery [1]. SSIs are associated with sepsis, increased mortality, prolonged hospital stays, higher healthcare costs, and other adverse outcomes. Several studies were conducted in different countries aiming to investigate the prevalence and the etiology of SSI. These studies showed a fluctuation in the prevalence in countries, from 0.67% in India, to 3.5% in Somalia, and 10.9% in Rwanda [1–3]. In Lebanon information about the prevalence of SSI are scare, in fact just one study highlights on the prevalence of SSI in Lower Extremity Amputation in diabetic patients which showed a rate of 6.8% among these patients [4].

Concerning the etiology of SSI, several studies showed that aerobically bacteria represent the main cause of this infection with *E. coli*, *K. pneumoniae* and *S. aureus* being the leading ones [2,3].

Management of infections at surgical sites is further complicated by the emergence of antibiotic-resistant pathogens, posing serious threats to both public health and individual patients [5].The issue of the rise of antibiotic resistance along with SSIs appears to be persistent in many countries, including Lebanon [6]. Antimicrobial resistance (AMR) represents a major challenge to modern healthcare systems worldwide, with studies documenting increasing resistance among key bacterial pathogens across diverse regions [7]. Excessive and inappropriate use of broad-spectrum antibiotics contributes to the development of resistance patterns in common bacterial pathogens. Salam et al. highlighted that AMR is a major public health threat, negatively affecting patient outcomes and increasing medical costs [8]. In Lebanon, data regarding the prevalence of SSIs and their associated antibiotic resistance remain limited, particularly in multiple hospital settings across the country [9].

Advanced diagnostic methodologies for identifying pathogenic bacteria and the antimicrobial resistance such as molecular diagnosis are extremely important, proving assets in epidemiological and clinical research. One critical concern for healthcare systems is the dissemination of antibiotic resistance genes (ARGs) among bacterial pathogens. Infections caused by multi-drug resistant organisms (MDROs) are associated with longer hospital stays, increased mortality, and higher rates of treatment failure [10].

Antibiotic resistant genes continue to be generated, and microbiology researchers are working on to develop new antibiotic classes. Among emerging research approaches, scientists now study bacterial metallophores as a way to create new antibiotic classes [11]. One example of research on ‘Trojan horse’ antibiotic delivery systems shows how they could break bacterial resistance mechanisms because they facilitate penetration of the antimicrobial agent into cells [12]. This study aims to determine the distribution of bacterial pathogens and evaluate their antibiotic profile among patients with SSIs in various Lebanese hospitals. This study gathered epidemiological data from different medical centers to clarify the current situation of bacterial SSIs and help design better infection control measures.

## Methods

### Study design

This research followed a multi-center and prospective cross-sectional study design, conducted across multiple hospitals from various regions in Lebanon, including Tyre, Beirut, Nabatieh, Saida, and Mount Lebanon. This study was conducted in the period between January 2024 and September 2024.

### Ethics statement

The Ethical approval was sought from the ethics committee and review board of the different hospitals (Al-Zahraa hospital (reference number: 7/2024), Sheikh Ragheb Harb Hospital (reference number: 10/2023), Al-Sahel hospital (reference number: 5/2024), Hammoud Hospital (reference number: 4/2024).

Al-Hayat Hospital and Italy Hospital do not have an ethical committee, we obtained approval through a signed permission letter sent from our university. In Jabal Amel Hospital, we received verbal approval, as they do not have an ethical committee.

Informed consent was obtained from all participants before sample collection. Written consent was obtained from patients in hospitals with an ethical committee. In hospitals without an ethical committee, Verbal consent was obtained and was documented in the medical records by the attending physician or study investigator.

### Study population and sample size

During the period of our study, 6933 patients underwent surgery in the participating seven hospital centers. These hospitals were private institutions with bed capacities ranging from 100 to 315. Out of the total number of patients, 63 cases were diagnosed with SSI based on the criteria established by the World Health Organization (WHO, 2016) and the Centers for Disease Control and Prevention (CDC, 2017), which include signs of infection at the surgical site such as redness, swelling, pain, purulent discharge, or delayed wound healing. Additionally, deep infections were identified through abscess formation, spontaneous wound dehiscence, or positive bacterial cultures from wound swabs or tissue samples. Organ/space SSIs were confirmed using imaging evidence of infection or microbiological testing [13,14].

### Sample collection and patient assessment

All relevant demographic and clinical data were obtained from patients’ medical records. The demographic variables included age, gender, nationality, educational level, occupational status, and blood type. Clinical information collected comprised type of admission, the surgical department performing the procedure, the nature and classification of the surgery, wound classification, and duration of the surgical procedure. Additional variables included type and timing of infection onset, prophylactic antibiotic use, and length of hospital stay. Patients’ medical history was also recorded, including diabetes, hypertension, cardiovascular diseases, and other chronic comorbidities. Samples were collected from Al-Zahraa Hospital starting on 10/3/2024, Sheikh Ragheb Harb Hospital on 2/1/2024, Al-Sahel Hospital on 30/3/2024, Hammoud Hospital on 8/3/2024, Al-Hayat Hospital on 27/2/2024, Jabal Amel Hospital on 1/2/2024, and Italy Hospital from 5/1/2024–29/9/2024 across all hospitals.

### Laboratory procedures

For analysis, the samples collected using a sterile swab from infected surgical sites were promptly transported to the microbiology laboratory. Those samples underwent the following procedures:

### Bacterial culture and identification

All specimens were cultured on Blood agar and Macconkey agar within one hour of collection. The inoculated agar plates were incubated aerobically at a temperature of 35–37°C. This study focused on aerobic bacterial pathogens, which are the most commonly associated with surgical site infections; anaerobic bacteria were not cultured as they represent a small proportion of SSI cases. After a period of 24 hours, the plates were examined for the presence of bacterial growth. If no growth is observed, the plates were incubated for an additional 24 hours.

The bacterial isolates that grew on the agar plates were identified through a series of standard microbiological techniques. This includes the morphology of colonies and Gram staining to classify the bacteria as either Gram-positive or Gram-negative. Further identification was achieved through different biochemical tests. For Gram-positive bacteria, tests such as catalase, coagulase, oxidase, and mannitol fermentation were conducted. For Gram-negative bacteria, tests including urease, indole, citrate, and sugar utilization were performed.

### Antibiotic susceptibility testing

The antibiotic susceptibility of bacterial isolates was evaluated using the Kirby-Bauer disk diffusion method, adhering to the guidelines of the Clinical Laboratory Standards Institute (CLSI). Selected bacterial colonies (3 –5) from a pure culture were transferred into a tube containing 5 mL of sterile distilled water and mixed gently until the turbidity matched the 0.5 McFarland standard.

A sterile cotton swab was used to evenly spread the bacteria across the surface of Mueller-Hinton agar. The plates were left to air-dry at room temperature for 3–5 minutes before placing antibiotic discs on the agar surface. Both Gram positive and Gram negative isolates was tested against: ampicillin (10 µg), gentamicin (10 µg), ciprofloxacin (5 µg), cefotaxime (30 µg), cefepime (30 µg), tetracycline (30 µg), trimethoprim-sulfamethoxazole (1.25/23.75 µg), cefoxitin (30 µg), fosfomycin (200 µg), tigecyline (15 µg). Gram-positive isolates were tested against penicillin (10 units), vancomycin (30 µg), erythromycin (15 µg), clindamycin (30 µg), oxacillin (5 µg), ceftriaxone (30 μg), teicoplanin (30 µg), rifampin (5 µg) and linezolid (30 µg). Gram negative isolates was tested against: amikacin (30 µg), ceftazidime (30 µg), cefuroxime (30 µg), amoxycillin + clavulanate (20/10 μg), aztreonam (30 µg), meropenem (10 µg), imipenem (10 µg), ertapenem (30 µg), piperacillin- tazobactam (100/10 µg), cefixime (5 µg), ceftolozane- tazobactam (40 µg) and ceftazidine-avibactam (30/20 µg). The plates were then incubated at 35–37°C for 18–24 hours.

After incubation, the diameter of the zones of inhibition around the antibiotic discs was measured, and the results were interpreted as sensitive, intermediate, or resistant based on established protocols by CLSI [15].

The classification of resistant bacteria, including Extended-Spectrum Beta-Lactamase (ESBL), Carbapenem-Resistant *Enterobacteriaceae* (CRE), and Methicillin-Resistant *Staphylococcus aureus* (MRSA), was based on phenotypic methods, utilizing the VITEK automated system and antimicrobial susceptibility testing through the disk diffusion method according to CLSI guidelines (2024 edition). For ESBL detection, the combination disk method was performed using cephalosporin disks (ceftazidime and cefotaxime) alone and in combination with clavulanic acid. A ≥ 5 mm increase in the zone diameter for the combination disk compared to the cephalosporin disk alone was considered confirmatory for ESBL production, as recommended by CLSI. For CRE classification, isolates showing reduced susceptibility or resistance to at least one carbapenem (ertapenem, meropenem, or imipenem) were further tested using the modified Hodge test and/or carbapenem inactivation method (CIM) to confirm carbapenemase production, following CLSI protocols. For MRSA identification, cefoxitin (30 µg) disk diffusion was performed, and a zone diameter ≤21 mm was interpreted as methicillin resistance. Oxacillin disk diffusion was also used for confirmation in certain cases, as recommended by CLSI. Bacterial isolates were considered multidrug resistant (MDR) if they showed resistance to at least one agent in three or more antimicrobial categories, following the standardized definitions for acquired resistance described by Magiorakos et al. (2012) [16].

### Data analysis

The data collected during the study were carefully entered into the Statistical Package for Social Sciences (SPSS) version 26 by one dedicated researcher. After entering the data, statistical analysis was performed using SPSS. Various descriptive statistics were calculated, including average values and frequency counts of bacterial distribution and AMR profile, to provide a comprehensive overview of the data.

## Results

### General prevalence of SSI

Out of 6933 patients who underwent a surgery in the surgical departments from the seven participated hospitals in the present study, 63 patients were positive for SSI showing a prevalence of 0.91%, 95% CI [0.70%, 1.15%].

### Socio-demographic characteristics

Table 1 shows the socio-demographic characteristics of the 63 SSI patients included in the present study. Females accounted for 63.5% (40 patients, 95% CI: 51.61–75.39%) and males for 36.5% (23 patients, 95% CI: 24.7–49.6%). The mean age was 57 years (SD = 21, 95% CI: 51.82–62.17), with 53.2% of patients aged over 60 years.

**Table 1:** Socio-Demographic Characteristics.

Characteristics		Frequency	Percentage (%)	Mean (SD)	95% CI
**Gender**	Male	23	36.5		24.7%, 49.6%
	Female	40	63.5		51.61%, 75.39%
**Age**	< 15	2	3.2		
	15 - 30	4	6.5		
	31 - 45	14	22.6	57 (21)	51.82, 62.17 years
	46 - 60	9	14.5		
	61 - 75	18	29		
	>75	15	24.2		
**Educational level**	No formal education	22	34.9		23.8%, 48%
	Primary	21	33.3		22%, 46.3%
	Secondary	17	27		16.6%, 39.7%
	University	3	4.8		1%, 13.3%

Regarding educational level, most patients had no formal education (34.9%, 95% CI: 23.8–48%) or primary education (33.3%, 95% CI: 22–46.3%), while secondary education (27%, 95% CI: 16.6–39.7%) and university degrees (4.8%, 95% CI: 1–13.3%) were less frequent.

### Clinical characteristics

**Fig 1:**
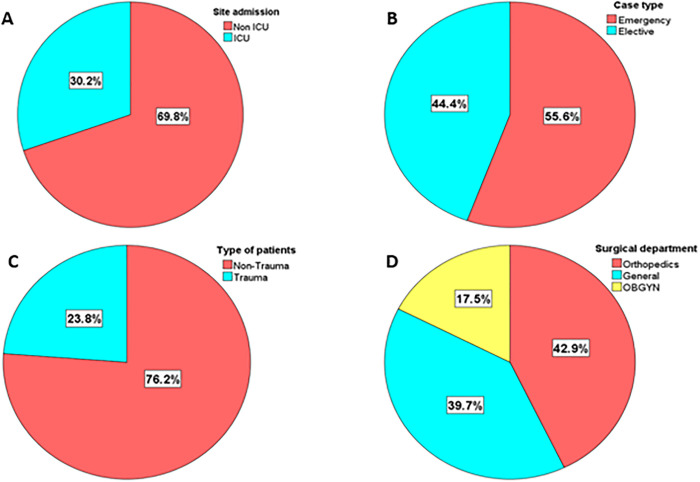
Clinical characteristics of SSIs Positive Patients. **A**, **B**, **C**, **D.**

Several parameters regarding the site of admission, the type of case, the type of patients and the surgical department were collected for patients (63 patients). According to Fig 1, 30.2%, 95% CI [19.2%, 43%] of participants were admitted to intensive care unit (ICU), whereas 69.8%, 95% CI [57%, 80.8%] were not (Fig 1A), 55.6%, 95% CI [42.5%, 68.1%] of patients were admitted to emergency, and only 44.4%, 95% CI [31.9%, 57.5%] with elective admission (Fig 1B), 23.8%, 95% CI [14%, 36.2%] of patients were admitted with trauma, while 76.2%, 95% CI [63.8%, 86%] not (Fig 1C). Finally, when studying the reason of admission to the hospital, our data show that 42.9%, 95% CI [30.5%, 56%] of patients were admitted to the hospital due to orthopedics surgeries, 39.7%, 95% CI [27.6%, 52.8%] due to general surgeries, and 17.5%, 95% CI [9.1%, 29.1%] due to Obstetrics and Gynecology (OBGYN) surgeries (Fig 1D).

Regarding the type of surgical procedure undergone by the SSI-positive patients’ cohort, Table 2 shows that the highest percentage was for C-Section surgery (17.9%), 95% CI [9.1%, 29.1%], followed by hip replacement, toe amputation, and drainage of abscess, with percentages ranging between 7.9%, 95% CI [2.6%, 17.6%], and 9.5%, 95% CI [3.6%, 19.6%]. Furthermore, hysterectomy, appendectomy, colectomy, laparotomy, removal of prosthesis, and other surgeries were reported at the lowest percentages (less than 5%).

**Table 2:** Types of Surgery Procedure underwent by Positive SSI patients.

Surgical Procedure	Number of Cases (n)	Percentage (%)	95% CI (%)
C-Section	11	17.9	9.1 – 29.1
Amputation of toes in foot	6	9.5	3.6 – 19.6
Drainage of abscess	5	7.9	2.6 – 17.6
Hip prostheses replacement	5	7.9	2.6 – 17.6
Laparotomy	3	4.8	1.0 – 13.3
Gastrectomy	3	4.8	1.0 – 13.3
Colectomy	3	4.8	1.0 – 13.3
Cataract	3	4.8	1.0 – 13.3
Whipple	3	4.8	1.0 – 13.3
Reduction of Hip	3	4.8	1.0 – 13.3
Cardiac catheterization	2	3.2	0.4 – 11.2
Leg amputation	2	3.2	0.4 – 11.2
Debridement	2	3.2	0.4 – 11.2
Laminectomy	2	3.2	0.4 – 11.2
Hysterectomy	1	1.6	0.0 – 8.6
Bones in brain	1	1.6	0.0 – 8.6
Hair transplant	1	1.6	0.0 – 8.6
Removal of Knee Prosthesis	1	1.6	0.0 – 8.6
Appendectomy	1	1.6	0.0 – 8.6
Lumbosacral spine	1	1.6	0.0 – 8.6
Axillary lymph node excision	1	1.6	0.0 – 8.6
Biliary atresia type 2	1	1.6	0.0 – 8.6
Polysite insertion	1	1.6	0.0 – 8.6
Excision of infected pilonidal cyst	1	1.6	0.0 – 8.6

**Fig 2:**
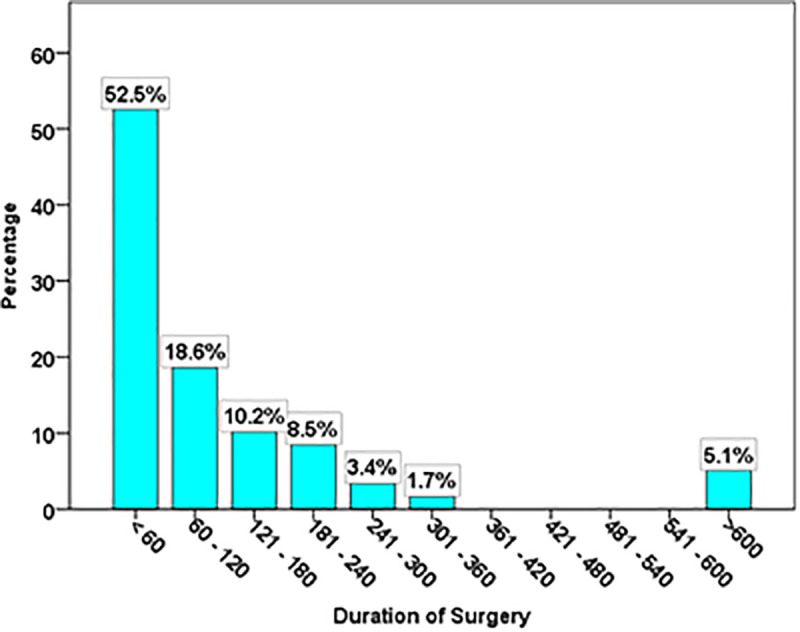
Duration of Surgery underwent by positive SSI patients.

Regarding the duration of surgery undergone by positive SSI patients, Fig 2 reports that most surgeries are of short duration, being done in less than one hour (52.5%),95% CI [36.4%, 62.1%], followed by 60–120 minutes (18.6%, 95% CI [9.1%, 29.1%]). Furthermore, the duration lasting between 121–180 minutes reported a low percentage (10.2%, 95% CI [93.6%, 19.6%]) to fall between 2–3 hours, whereas, 181–240 minutes also reported a low percentage (8.5%, 95% CI [2.6%, 17.6%]) to fall between 3–4 hours. However, longer durations were reported to gradually decrease for 241–300 minutes (3.4%, 95% CI [0.4%, 11%]) and for 301–360 minutes (1.7%, 95% CI [0%, 8.5%]). Remarkably, there is a (5.1%, 95% CI [1%, 13.3%]) for surgeries that take more than 600 minutes, which could be related to complex or specialized surgeries.

Table 3 reported that the SSI classification was reported for 63.5% as Superficial SSI in 40 patients, 95% CI [50.4%, 75.3%], and 36.5%, 95% CI [24.7%, 49.6%] as Deep SSI in 23 patients. As for the wound classification, 47 patients have a clean wound classification (74.6%), 95% CI [62.1%, 84.7%], followed by Clean–Contaminated classification (12.7%, 95% CI [5.6%, 23.5%]), and finally both the contaminated wound and the dirty-infected groups with a percentage of 6.3%, 95% CI [1.8%, 15.5%]. Accordingly, regarding the antibiotic prophylaxis, only 31.75%, 95% CI [20.6%, 44.7%] received prophylaxis (20.6%, 95% CI [11.5%, 32.7%] Preoperative, 11.1%, 95% CI [4.6%, 21.6%] at the beginning of the surgery - Perioperative), compared to 68.3% who did not receive any antibiotic prophylaxis, 95% CI [55.3%, 79.4%], being the highest percentage.

**Table 3:** Characteristics of Infection in SSI positive Patients.

Characteristics		Frequency	Percentage (%)	95% CI
Surgical site infection class	Superficial	40	63.5	50.4%, 75.3%
	Deep	23	36.5	24.7%, 49.6%
Wound classification	Clean	47	74.6	62.1%, 84.7%
	Contaminated	4	6.3	1.8%, 15.5%
	Clean-Contaminated	8	12.7	5.6%, 23.5%
	Dirty-Infected	4	6.3	1.8%, 15.5%
Antibiotic prophylaxis	Preoperative	13	20.6	11.5%, 32.7%
	Postoperative	0	0	
	At the beginning	7	11.1	4.6%, 21.6%
	Not at all	43	68.3	55.3%, 79.4%

### Bacterial Isolates

Microbiological identification results show that the prevalence of Gram – negative bacteria was higher than that of Gram-positive bacteria (73% “95% CI [60.3%, 83.4%]” VS 27%, 95% CI [16.6%, 39.7%]) as shown in Table 4. Regarding bacterial species, *Escherichia coli* (*E. coli*) was the most prevalent isolated bacterium from SSI patients (20.6%, 95% CI [11.5%, 32.7%]) followed by *Pseudomonas aeruginosa* (*P. aeruginosa*) (19%, 95% CI [10.2%, 30.9%]), *Klebsiella pneumonia* (*K. pneumoniae*) (15.9%, 95% CI [7.9%, 27.3%]), *Staphylococcus aureus* (*S. aureus*) (12.7%, 95% CI [5.6%, 23.5%]) *Acinetobacter baumannii* (*A. baumannii*) (9.5%, 95% CI [3.6%, 19.6%]), *Staphylococcus Coagulase negative* (*CoNS*) (9.5%, 95% CI [3.6%, 19.6%]) accounting for 87.2%, 95% CI [76.5%, 94.4%] of all isolates (Table 4). All other bacterial species (*Streptococcus*, *Enterococcus*, *Proteus mirabilis* (*P. mirabilis*), and *Enterobacter cloacae*) were detected in 12.8%, 95% CI [5.6%, 23.5%] of patients (Table 4).

**Table 4:** Bacterial Isolates.

Bacterial isolate	Frequency	Percentage (%)	95% CI
*Gram Positive*	**17**	**27%**	16.6%, 39.7%
			
*Staphylococcus aureus*	8	12.7%	5.6%, 23.5%
*Streptococcus*	1	1.6%	0%, 8.5%
*Enterococcus*	2	3.2%	0.4%, 11%
*Staphylococcus Coagulase Negative*	6	9.5%	3.6%, 19.6%
*Gram Negative*	**46**	**73%**	60.3%, 83.4%
*Escherichia coli*	13	20.6%	11.5%, 32.7%
*Klebsiella pneumoniae*	10	15.9%	7.9%, 27.3%
*Acinetobacter baumannii*	6	9.5%	3.6%, 19.6%
*Pseudomonas aeruginosa*	12	19%	10.2%, 30.9%
*Proteus mirabilis*	4	6.3%	1.8%, 15.5%
*Enterobacter cloacae*	1	1.6%	0%, 8.5%
*Total*	*63*	*100%*	

Regarding the distribution of bacterial pathogens with respect to the type of SSI (Superficial or Deep), Table 5 shows that the most common bacteria found in superficial SSIs were E. coli (20%),95% CI [9.1%, 35.6%], followed by S. aureus and K. pneumoniae (both at 17.5%),95% CI [7.3%, 32.8%]. A. baumannii and P. aeruginosa each accounted for 12.5%, 95% CI [4.2%, 26.8%] of superficial SSIs. P. mirabilis had a frequency of 10%, 95% CI [2.8%, 23.7%], while CoNS was responsible for 5%, 95% CI 0.6%, 16.9%].

**Table 5:** Frequency of Bacterial Isolates in Superficial and Deep Tissue Surgical Site Infections (SSIs).

Bacterial Isolate	Superficial SSI: Frequency (%)	95% CI	Deep SSI: Frequency (%)	95% CI	Total (%)
Escherichia coli	8 (20)	9.1%, 35.6%	5 (21.7)	7.5%, 43.7%	**13 (20.6)**
Pseudomonas aeruginosa	5 (12.5)	4.2%, 26.8%	7 (30.4)	13.2%, 52.9%	**12 (19)**
Klebsiella pneumoniae	7 (17.5)	7.3%, 32.8%	3 (13)	2.8%, 33.6%	**10 (15.9)**
Staphylococcus aureus	7 (17.5)	7.3%, 32.8%	1 (4.3)	0.1%, 21.9%	**8 (12.7)**
Acinetobacter baumannii	5 (12.5)	4.2%, 26.8%	1 (4.3)	0.1%, 21.9%	**6 (9.5)**
Staphylococcus Coagulase-Negative	2 (5)	0.6%, 16.9%	4 (17.4)	5%, 38.8%	**6 (9.5)**
Proteus mirabilis	4 (10)	2.8%, 23.7%	0 (0)	—	**4 (6.3)**
Enterococcus spp.	0 (0)	—	2 (8.7)	1.1%, 28%	**2 (3.2)**
Streptococcus spp.	1 (2.5)	0.1%, 13.2%	0 (0)	—	**1 (1.6)**
Enterobacter cloacae	1 (2.5)	0.1%, 13.2%	0 (0)	—	**1 (1.6)**

For deep tissue SSIs, P. aeruginosa was the most prevalent bacterium, accounting for 30.4% of cases, 95% CI [13.2%, 52.9%]. It was followed by E. coli at 21.7%, 95% CI [7.5%, 43.7%] and CoNS at 17.4%, 95% CI [5%, 38.8%]. S. aureus made up 4.3%, 95% CI [0.1%, 21.9%] of deep tissue SSIs. Other bacteria, including Streptococcus, P. mirabilis, and Enterococcus, had lower frequencies, all reported in only 8.7%, 95% CI [1.1%, 28%] of cases.

### Antibiotic resistance profile

#### Antibiotic susceptibility of Gram-negative isolates.

Antibiotic susceptibility among Gram-negative isolates showed marked variability across antibiotic classes (Table 6). Penicillins demonstrated limited activity, with resistance to ampicillin reaching 100% in *K. pneumoniae, A. baumannii, P. mirabilis,* and *E. cloacae*, while only 46.2% of *E. coli* isolates were sensitive. Piperacillin–tazobactam showed better performance, particularly against *P. aeruginosa* (80% sensitivity) and *E. coli* (72.7%), but was less effective against *K. pneumoniae* (30%) and *A. baumannii* (25%).

**Table 6:** Antibiotic susceptibility of Gram-Negative isolates.

Antimicrobial agents	Pattern	Bacterial isolate					
		*E. coli*	*K. pneumoniae*	*A. baumannii*	*P. aeruginosa*	*P. mirabilis*	*E. cloacae*
AMP	Sensitive	46.2	0	0	60	0	0
	Resistant	53.8	100	100	40	100	100
TZP	Sensitive	72.7	30	25	80	75	0
	Resistant	27.3	60	75	0	25	100
AMC	Sensitive	46.2	20	0	55.6	25	0
	Resistant	46.2	80	100	44.4	50	100
CXM	Sensitive	46.2	30	33.3	58.3	25	0
	Resistant	53.8	70	66.7	41.7	75	100
FOX	Sensitive	61.5	20	16.7	58.3	25	0
	Resist	38.5	80	83.3	41.7	75	100
CTX	Sensitive	46.2	20	16.7	58.3	25	0
	Resistant	53.8	80	83.3	41.7	75	100
CFM	Sensitive	46.2	20	16.7	58.3	25	0
	Resistant	53.8	80	83.3	41.7	75	100
CAZ	Sensitive	53.8	20	16.7	58.3	75	0
	Resistant	46.2	80	66.7	41.7	25	100
MEM	Sensitive	84.6	80	33.3	100	75	0
	Resistant	15.4	20	66.7	0	25	100
IMP	Sensitive	84.6	70	33.3	100	50	0
	Resistant	15.4	20	66.7	0	25	100
ETP	Sensitive	84.6	80	33.3	100	75	0
	Resistant	15.4	20	66.7	0	25	100
AN	Sensitive	100	80	16.7	100	75	0
	Resistant	0	0	83.3	0	25	0
GM	Sensitive	76.9	70	16.7	91.7	75	0
	Resistant	23.1	20	83.3	8.3	25	0
CIP	Sensitive	61.5	60	33.3	75	66.7	0
	Resistant	38.5	40	66.7	25	33.3	100
LEV	Sensitive	61.5	50	33.3	75	50	0
	Resistant	38.5	50	66.7	25	50	100
SXT	Sensitive	69.2	40	20	70	0	0
	Resistant	30.8	60	80	30	100	100
FEP	Sensitive	69.2	60	33.3	75	75	0
	Resistant	30.8	40	66.7	25	0	100
TET	Sensitive	50	33.3	0	100	0	0
	Resistant	50	66.7	100	0	0	100
ATM	Sensitive	61.5	37.5	25	75	100	0
	Resistant	30.8	62.5	75	25	0	100
FOS	Sensitive	100	40	0	100	0	0
	Resistant	0	60	100	0	0	100
TIG	Sensitive	62.5	57.1	80	0	0	0
	Resistant	37.5	42.9	20	0	0	100
CT	Sensitive	100	50	0	100	100	0
	Resist	0	50	0	0	0	0
CZA	Sensitive	81.8	90	50	100	100	0
	Resistant	0	50	0	0	0	0

Ampicillin (AMP), Piperacillin-Tazobactam (TZP), Amoxicillin-clavulanate (AMC), Cefuroxime (CXM), Cefoxitin (FOX), Cefotaxime (CTX), Cefixime (CFM), Ceftazidine (CAZ), Meropenem (MEM), Imipenem (IMP), Ertapenem (ETP), Amikacin (AN), Gentamicin (GM), Ciprofloxacin (CIP), Levofloxacin (LEV), Trimethroprim-sulfamethoxazole (SXT), Cefepime (FEP), Tetracycline (TET), Aztreonam (ATM), Fosfomycin (FOS), Tigecyline (TIG), Ceftolozane- Tazobactam (CT), Cftazidine- Avibactam (CZA).

Cephalosporins showed moderate susceptibility in *E. coli* (46.2–53.8%) and *P. aeruginosa* (58.3%), whereas *A. baumannii* and *E. cloacae* remained highly resistant (≥66.7% resistance across most agents). Carbapenems demonstrated high activity against *E. coli* (84.6%) and *P. aeruginosa* (100%), with reduced effectiveness seen in *A. baumannii* (33.3%) and *E. cloacae* (0%).

Aminoglycosides showed strong activity, particularly amikacin (100% in *E. coli* and *P. aeruginosa* and 80% in *K. pneumoniae*), while susceptibility in *A. baumannii* was low (16.7%). Fluoroquinolone sensitivity ranged from 50–75% in most species but remained low in *A. baumannii* (33.3%) and absent in *E. cloacae* (0%).

Newer therapeutic options performed best: ceftazidime–avibactam and Ceftolozane- Tazobactam showed high sensitivity in *E. coli* and *P. aeruginosa*, while susceptibility in *K. pneumoniae* ranged from 50–90%. However, *E. cloacae* remained fully resistant to most agents.

### Antibiotic susceptibility of gram-positive isolates

Gram-positive isolates showed high susceptibility to key agents (Table 7). Vancomycin demonstrated 100% activity against all isolates, including *S. aureus*, *CoNS, Streptococcus, and Enterococcus*. Linezolid showed similar activity (100% sensitivity) except in *CoNS* (66.7%). Tigecycline was highly active, with sensitivity ranging from 85.7% in *S. aureus* to 100% in all other Gram-positive species.

**Table 7:** Antibiotic susceptibility of Gram-positive isolates.

Antimicrobial agents	Pattern	Bacterial isolate			
		Staphylococcus aureus	Staphylococcus Coagulase Negative	Streptococcus	Enterococcus
FOX	Sensitive	37.5	40	100	0
	Resistant	62.5	60	0	0
Oxa	Sensitive	42.9	16.7	0	50
	Resistant	57.1	83.3	0	50
AMP	Sensitive	50	20	100	100
	Resist	50	80	0	0
P	Sensitive	25	25	0	100
	Resistant	75	75	0	0
E	Sensitive	37.5	16.7	0	50
	Resistant	62.5	83.3	0	50
CC	Sensitive	50	50	100	100
	Resist	50	50	0	0
Van	Sensitive	100	100	100	100
	Resistant	0	0	0	0
Tec	Sensitive	87.5	100	0	100
	Resistant	0	0	0	0
SXT	Sensitive	87.5	66.7	100	100
	Resistant	12.5	33.3	0	0
Rif	Sensitive	100	0	0	0
	Resistant	0	0	0	0
GM	Sensitive	100	33.3	100	50
	Resistant	0	66.7	0	50
LIN	Sensitive	100	66.7	100	100
	Resistant	0	33.3	0	0
TET	Sensitive	87.5	33.3	100	0
	Resistant	12.5	66.7	0	100
FEP	Sensitive	42.9	50	100	0
	Resistant	57.1	50	0	0
CTX	Sensitive	33.3	50	100	0
	Resistant	66.7	50	0	0
CRO	Sensitive	42.9	50	100	0
	Resistant	57.1	50	0	0
CIP	Sensitive	37.5	16.7	100	100
	Resistant	62.5	83.3	0	0
LEV	Sensitive	37.5	16.7	100	100
	Resistant	62.5	83.3	0	0
FOS	Sensitive	0	0	0	0
	Resistant	0	100	0	0
TIG	Sensitive	85.7	100	100	100
	Resistant	14.3	0	0	0

Cefoxitin (FOX), Oxacillin (Oxa), Ampicillin (AMP), Penicillin (P), Erythromycin (E), Clindamycin (CC), Vancomycin (Van), Teicoplanin (Tec), Trimethroprim-Sulfamethoxazole (SXT), Rifampin (Rif), Gentamicin (GM), Linezolid (LIN), Tetracycline (TET), Cefepime (FEP), Cefotaxime (CTX), Ceftriaxone (CRO), Ciprofloxacin (CIP), Levofloxacin (LEV), Fosfomycin (FOS), Tigecycline (TG).

In contrast, β-lactam susceptibility was lower. Oxacillin sensitivity was 42.9% in *S. aureus* and 16.7% in *CoNS*, while ampicillin remained fully active against *Streptococcus* and *Enterococcus* (100%). Macrolides demonstrated limited effectiveness, with erythromycin sensitivity dropping to 37.5% in *S. aureus* and 16.7% in *CoNS.*

Clindamycin and trimethoprim–sulfamethoxazole showed moderate to high activity, with sensitivity ranging 50–100% depending on the species. Gentamicin was fully active against S. aureus and Streptococcus (100%) but lower in CoNS (33.3%) and Enterococcus (50%). Cephalosporin resistance was notable in S. aureus and CoNS (up to 66.7% resistance), whereas Streptococcus remained fully sensitive (100%).

### Resistance of bacterial isolates

Among the bacterial isolates, 27% were Gram-positive and 73% were Gram-negative (Table 8).

**Table 8:** Resistance of Bacterial Isolates.

Bacterial isolate	Total number	Antimicrobial resistant pattern: Total number (%)						
		R0	R1	R2	R3	R4	R5	R6
***Gram Positive***	17	2 (11.8)	1 (5.9)	2 (11.8)	2 (11.8)	5 (29.4)	3 (17.6)	2 (11.8)
*S. aureus*	8	1 (12.5)	0 (0)	1 (12.5)	0 (0)	3 (37.5)	3 (37.5)	0 (0)
*CoNS*	6	0 (0)	1 (16.7)	0 (0)	1 (16.7)	2 (33.3)	0 (0)	2 (33.3)
*Streptococcus*	1	1 (100)	0 (0)	0 (0)	0 (0)	0 (0)	0 (0)	0 (0)
*Enterococcus*	2	0 (0)	0 (0)	1 (50)	1 (50)	0 (0)	0 (0)	0 (0)
***Gram Negative***	46	8 (17.4)	5 (10.9)	5 (10.9)	5 (10.9)	11 (23.9)	7 (15.2)	5 (10.9)
*E. coli*	13	4 (30.8)	0 (0)	4 (30.8)	1 (7.7)	2 (15.4)	1 (7.7)	1 (7.7)
*K. pneumonia*	10	0 (0)	2 (20)	0 (0)	1 (10)	5 (50)	0 (0)	2 (20)
*A. baumannii*	6	0 (0)	1 (16.7)	0 (0)	1 (16.7)	0 (0)	3 (50)	1 (16.7)
*P. aeruginosa*	12	4 (33.3)	2 (16.7)	1 (8.3)	1 (8.3)	3 (25)	1 (8.3)	0 (0)
*P. mirabilis*	4	0 (0)	0 (0)	0 (0)	1 (25)	1 (25)	2 (50)	0 (0)
*E. cloacae*	1	0 (0)	0 (0)	0 (0)	0 (0)	0 (0)	0 (0)	1 (100)

R_0_: no resistance, R_1_: resistance to one drug, R_2_: resistance to two drugs, R_3_: resistance to three drugs, R_4_: resistance to four drugs, R_5_: resistance to five drugs, R_6_: resistance to six drugs.

### Gram-positive isolates

Most Gram-positive bacteria exhibited high levels of multidrug resistance (MDR). Overall, 70.6% of Gram-positive isolates were MDR. *S. aureus* showed 75% MDR, while *CoNS* presented 83.3% MDR. In contrast, *Streptococcus* isolates showed no resistance, and *Enterococcus* displayed moderate resistance, with 50% MDR.

### Gram-negative isolates

Among Gram-negative isolates, 60.9% were identified as MDR strains. *Klebsiella pneumoniae* exhibited 80% MDR, *A. baumannii* 83.3% MDR, *P. aeruginosa* 41.7% MDR, and *P.mirabilis* 100% MDR. *E. coli* showed a lower MDR rate (38.5%), whereas *Enterobacter cloacae* was entirely resistant (100% MDR). Detailed resistance patterns are presented in Table 8.

### Specific resistance strains

Regarding some specific resistant strains, we analyzed the frequency of Extended Spectrum Beta-Lactamase (ESBL) producing bacterial isolates, Carbapenem-resistant Enterobacteriaceae (CRE), and Methicillin-resistant *Staphylococcus aureus* (MRSA). Table 9 shows that Enterobacter cloacae reported the highest frequency of ESBL production, with 1 isolate (100%, 95% CI [20.7%, 100.0%]) being an ESBL producer. Among the other isolates, *K. pneumoniae* reported a high proportion of ESBL production with 8 isolates (80.0%, 95% CI [49.0%, 94.3%]), followed by *E. coli* with 7 isolates (53.8%, 95% CI [29.1%, 76.8%]). *P. mirabilis* and *A. baumannii* each had an equal distribution of ESBL and non-ESBL isolates, with 2 isolates (50.0%, 95% CI [15.0%, 85.0%]) and 3 isolates (50.0%, 95% CI [18.8%, 81.2%]) respectively.

**Table 9:** Distribution of ESBL-Producing Bacterial Isolates.

Bacterial isolate	Non-ESBL: frequency (%)	95% CI	ESBL: frequency (%)	95% CI
***Escherichia coli***	6 (46.2%)		7(53.8%)	29.1%, 76.8%
***Klebsiella pneumonia***	2 (20%)		8 (80%)	49%, 94.3%
***Acinetobacter baumannii***	3 (50%)		3 (50%)	18.8%, 81.2%
				
***Proteus mirabilis***	2 (50%)		2 (50%)	15%, 85%
***Enterobacter cloacae***	0 (0%)		1 (100%)	
***Total***	13 (38.2%)	23.9%, 55%	21 (61.8%)	45%, 76.1%

Overall, out of 34 total isolates, 21 (61.8%, 95% CI [45.0%, 76.1%]) were ESBL producers, and 13 (38.2%, 95% CI [23.9%, 55.0%]) were non-ESBL producers. This indicates that ESBL-producing isolates are more prevalent in the studied sample, highlighting a significant presence of antibiotic-resistant bacteria.

Table 10 shows that, for the *S. aureus* isolates, 3 isolates (37.5%, 95% CI [8.5%, 75.5%]) were non-MRSA, while 5 isolates (62.5%, 95% CI [44.4%, 97.5%]) were MRSA.

**Table 10:** Distribution of MRSA Isolates in Staphylococcus aureus.

Bacterial isolate	Non-MRSA: Frequency (%)	95% CI	MRSA: Frequency (%)	95% CI
***S aureus***	3 (37.5%)	8.5%, 75.5%	5 (62.5%)	44.4%, 97.5%]

Table 11 shows that *A. baumannii* reported the highest frequency of CR, with 4 isolates (66.7%, 95% CI [23.3%, 95.7%]) being CR. For *E. coli*, 2 isolates (15.4%, 95% CI [1.9%, 45.4%]) were CR, while *K. pneumoniae* had 2 isolates (20%, 95% CI [2.5%, 55.6%]) that were CR. *P. aeruginosa* had no CR isolates (0%), and *E. cloacae* had 1 isolate (100%) that was CR. Overall, out of 42 isolates, 9 (21.4%, 95% CI [10.3%, 36.8%]) were CR, and 33 (78.6%, 95% CI [63.2%, 89.7%]) were non-CR.

**Table 11:** Frequency of carbapenem resistance in Various Bacterial Species.

Bacterial isolate	Non-CR-GN: Frequency (%)	95% CI	CR-GN: Frequency (%)	95% CI
***Escherichia coli***	11 (84.6%)		2 (15.4%)	1.9%, 45.4%
***Klebsiella pneumoniae***	8 (80%)		2 (20%)	2.5%, 55.6%
***Acinetobacter baumannii***	2 (33.3%)		4 (66.7%)	23.3%, 95.7%
***Pseudomonas aeruginosa***	12 (100%)		0 (0%)	
***Enterobacter cloacae***	0 (0%)		1 (100%)	
***Total***	33 (78.6%)	63.2%, 89.7%	9 (21.4%)	10.3%, 36.8%

## Discussion

The prevalence of SSI in our study was 0.91%, which is notably lower than the rates reported in other studies, such as the 10.9% incidence observed in a tertiary care hospital in Rwanda, 3.5% in Mogadishu Somalia Turkish Training and Research Hospital, 0.67% in Ahmedabad, Gujarat, India [2,3,17]. This difference may be explained by variations in infection control policies, surgical practices, and antibiotic stewardship programs across healthcare systems. Moreover, the absence of post-discharge surveillance in our study may have led to an underestimation of SSI prevalence, as infections occurring after discharge could not be captured. For instance, the Rwanda study highlighted prolonged hospital stays, emergency surgeries, and inadequate antibiotic prophylaxis as significant risk factors, which align with findings in our study, where 68.3% of patients did not receive antibiotic prophylaxis [2].

Compared to males in the present study, at 36.5%, there is a higher prevalence of SSI among females, with 63.5% of the cases. The study population had a mean age of 57 years (SD = 21), and the majority of cases were observed in patients aged 61–75 years (29%) and those over 75 years (24.2%), indicating more susceptibility in older populations. These findings differ from the study in Rwanda, which identified 13.3% for males and 7.7% for females [2]. Additionally, the educational level of patients in our study showed a majority with no formal education (34.9%), which may influence health literacy and adherence to postoperative care instructions, consistent with the study in Rwanda, where 55.8% of patients had primary education [2].

Regarding clinical characteristics, our study found that 42.9% of SSIs were associated with orthopedic surgeries, followed by general surgeries (39.7%). While the findings from the study on bacterial pathogens in tertiary care hospitals in Ahmedabad, Gujarat, India, reported a high prevalence of SSIs in abdominal surgeries (52.9%), followed by pelvic surgeries (14.71%) [17]. The duration of surgery was another critical factor, with most SSIs occurring in surgeries lasting less than one hour (52.5%). However, longer durations were associated with complex procedures, as noted in the Rwandan study (55.8%) [2]. Deep infections represented 36.5% of cases, while superficial SSIs had 63.5% in our study, which differs from the study in Ethiopia, where 57.9% were from deep infections and 42.1% were from superficial [1]. Clean wounds were the most common classification at 74.6%, followed by clean-contaminated wounds at 12.7%. The proportion of dirty-infected and contaminated wounds was at 6.3%. in comparison to the study conducted in Ahmedabad, Gujarat, India, clean surgeries were 0.7%, clean-contaminated 0.5%, contaminated 0.8%, and dirty wounds 1.3% [17]. In our study, 68.3% of patients didn’t received antibiotic prophylaxis before surgery while all patients received cephalosporin antimicrobial prophylaxis before the operation in the Mogadishu Somalia Turkish Training and Research Hospital [3]. This difference can be explained better if we got information about the type of surgery and the inclusions criteria in patients participating in the Somalian study.

The microbiological profile was dominated by Gram-negative bacteria (73%), with *E. coli*, *P. aeruginosa*, and *K. pneumoniae* being the most prevalent species. This predominance of Gram-negative pathogens is consistent with studies in Somalia and India, where *E. coli* and *S. aureus* were frequently reported [3]. The high prevalence of *E. coli* and *P. aeruginosa* reflects their known ability to form biofilms, secrete metallophores, and persist in the hospital environment, facilitating SSI development [18–20]. Similarly, the virulence of *S. aureus* and *CoNS* can be attributed to their biofilm-forming ability and expression of iron-acquisition systems such as metallophores [20]. Among Gram-positive isolates, the predominance of *S. aureus* (12.7%) mirrors global patterns and highlights its persistent role as a leading SSI pathogen. *Enterococcus* and *Streptococcus* species also contributed to infections, albeit at lower rates, reflecting their ability to survive harsh hospital conditions and transfer resistance genes [18,21].

Antibiotic resistance analysis revealed high resistance to β-lactams, particularly ampicillin and cefuroxime, among Gram-negative isolates, in agreement with studies in Somalia and Rwanda [3]. Carbapenems (meropenem, imipenem) and aminoglycosides (amikacin, gentamicin) remained the most effective agents against *E. coli* and *P. aeruginosa*, consistent with previous reports [2]. However, resistance to carbapenems among *A. baumannii* (66.7%) underscores its clinical importance as a critical-priority pathogen. For Gram-positive isolates, vancomycin and teicoplanin retained full effectiveness, in agreement with studies in Ethiopia and India [1,17]. Conversely, macrolides and fluoroquinolones showed high resistance in S. aureus and CoNS, similar to earlier findings [1].

Within the examination of the patterns of resistance, Multidrug-resistant (MDR) strains were prevalent in both Gram-positive and Gram-negative isolates, with 70.6% of Gram-positive and 60.9% of Gram-negative isolates classified as MDR. This aligns with the study in Ethiopia, which reported high rates of MDR strains [1]. Specific resistant strains such as ESBL-producing Enterobacteriaceae and MRSA were also identified, highlighting the global challenge of antimicrobial resistance [3,17]. ESBL-producing Enterobacteriaceae accounted for 56.5% of Gram-negative isolates, while MRSA was identified in 62.5% of *S, aureus* isolates. These figures closely align with global reports emphasizing the emergence of resistant strains in healthcare settings [17]. Carbapenem-resistant Enterobacteriaceae (CRE) were present in 21.4% of isolates, with *A. baumannii* exhibiting the highest CRE proportion (66.7%). In another study, the prevalence of ESBL was high (82.61%), MRSA and CRE were moderate, respectively (41.67%) and (43.48%) [17]. The presence of ESBL and MRSA further underscores the global challenge of antimicrobial resistance and the need for comprehensive stewardship programs. These programs aim to optimize antibiotic use through evidence-based prophylaxis, regular monitoring, and clinician education, which are essential to reducing resistance and improving patient outcomes.[22]

These findings have consequences for the conduct of active practice in Lebanese hospitals [23]. Whereas, there is a need to empirically choose antibiotics that would serve as treatment for the majority of the Gram-negative bacteria above. Hence, they estimate that these antibiotics may not cover this bacterium, and for that reason, there is a moderate power of multidrug bacterial resistance. However, the results are alarming; therefore, hospitals must use extreme caution when administering these antibiotics. On the other hand, the rate of antibiotic prophylaxis is low at 31.7%; hence, the above protocols are poor. In addition, it is crucial to include the fact that there is high primary multiple-drug resistance; this implies that culture-guided therapy is preferred. The findings of this study underscore the importance of implementing targeted infection control measures, optimizing antibiotic prophylaxis, and enhancing surveillance systems to mitigate the burden of SSIs. Future research should focus on exploring the genetic mechanisms underlying antimicrobial resistance and developing novel therapeutic strategies.

### Limitations

This study has several limitations that should be acknowledged. First, the absence of post-discharge surveillance may have resulted in an underestimation of the true prevalence of SSIs, as some infections may have developed after patients left the hospital and were therefore not captured during the study period. Second, anaerobic culturing was not performed, which may have led to missed identification of obligate anaerobes known to contribute to certain types of SSIs, particularly deep or organ-space infections.

## Conclusion

The overall incidence rate of surgical site infections (SSIs) was 0.91%, with Gram-negative bacteria (73%) predominating. The most common pathogens were *E. coli*, *P. aeruginosa*, and *K. pneumoniae*. High rates of multidrug resistance (MDR) were observed, particularly among *A. baumannii* (83.3%), *K. pneumoniae* (80%), and *P. mirabilis* (100%). Among Gram-positive isolates, *S. aureus* and *CoNS* showed MDR rates of 75% and 83.3%, respectively.

Carbapenems (meropenem, ertapenem, imipenem) were the most effective antibiotics against Gram-negative isolates, while vancomycin, teicoplanin, and linezolid were highly effective against Gram-positive bacteria. Ampicillin, cefuroxime, and amoxicillin–clavulanate demonstrated poor activity, especially against *K. pneumoniae* and *A. baumannii*.

The use of prophylactic antibiotic and associated clinical factors, such as advanced age and general or orthopedic surgeries, likely contributed to infection risk. These findings emphasize the need for stronger infection control policies, antimicrobial stewardship, and culture-based therapy to combat rising resistance trends.

## Supporting information

S1 Data Dataset supporting the findings of the study, including patient demographics, clinical characteristics, microbiological isolates and antibiotic susceptibility results. (SAV)
